# Nurse Democrats Voice Their Problems. Important Questions and Clear Answers

**Published:** 1917-07-21

**Authors:** Arthur Stanley


### THE COLLEGE OF NURSING, LIMITED.

### NURSE DEMOCRATS VOICE THEIR PROBLEMS.

### Important Questions and Clear Answers.

**The Hon. ARTHUR STANLEY, C.B., M.P.**

I have some questions here which have been either sent to me or handed up, and which I will try to deal with. The last time I tried to deal with a question in the presence of Miss Musson, down at Birmingham, I gave a wrong answer, but as she promptly contradicted me afterwards, we came all right at the end. (Laughter.)

An Elective Council.

A lady writes from the 1st London General Hospital:—"Would it not be possible for a certain proportion of the Council to retire immediately after the passing of the Bill for State Registration, without waiting for the period of two years to elapse as suggested?" Of course, when the Bill is passed the Council will have been set up by Parliament, and Parliament will make such regulations for the retiring in due course of members of the Council as they may think fit. But our one intention is to get this Council, under the Bill, elective as soon as we possibly can. There will always have to be a certain number of nominated members, nominated probably by the Privy Council, by the British Medical Association, and others; but so far as the College of Nursing is concerned, our aim and object is to get proper representation of the nurses by election as soon as we possibly can. If it is found possible to do this under two years, we certainly should not object.

Fellowship Branches.

Another lady asks:—"May I ask whether the Council have any intention of starting branches throughout the country for promoting fellowship, and, should such Branches be formed, what provision would be made for nurses in training?" Well, I think I have already dealt with that in the remarks which I made before. Wherever it is a good thing to have a branch, wherever the nurses in a district think it is advisable to have a branch, and think they are strong enough and numerous enough to form one, we shall be very glad to welcome it. In that connection I should just like to mention a resolution which was proposed by Miss Pilgrim, Inspector for the County of Lancashire, and seconded by Miss Smith, Matron of the Withington Hospitals, and carried unanimously:—"That this meeting of members of the College of Nursing, Limited, pledge themselves to help forward the interests of the College, especially in the matter of getting new members, and wish to express their confidence in the work of the Council of the College." That was passed under the presidency, I think, of Miss Sparshott, down at Manchester. If we get similar assistance from branches throughout the whole country, then it will very materially assist our work at headquarters. (Hear, hear.)

Children's Trained Nurses.

The next question is:—"Are nurses holding certificates as children's nurses to be on the same roll with those who hold certificates for general training?" What we are doing at the present moment is to form a Register of those nurses who have got general training—of those nurses who hold certificates as approved by the College for general training in recognised Training Schools. That is the foundation, of course, of the whole of the College. The Register of the College will be a Register of certificated trained nurses. Whether there will be Supplementary Registers afterwards for other branches of special training in nursing will be a matter which the College itself and the Council will have to decide.

A Benevolent Fund.

Another lady asks: "Will there be any bank nurses can join in connection with the College? Also a Pension Fund?" (Laughter.) Well, if the lady who asks that has got a sufficiently large balance of her own, we will certainly help her to open an account at our own bank. The Pension Fund is, of course, a different matter. There is already, as you know, the Royal National Pension Fund, which provides for pensions for nurses on recognised business principles. I have not said anything to-day about the fund which we hope to institute for trained nurses who have fallen on evil days, but that is one of the objects of the College, and I hope we shall very soon be able to work at it. (Applause.)

Protection for the General Trained Nurse.

Then there is another question which has been passed up. The first part is about a Children's Register, and that I have already dealt with. The last part of the question is: "Will it be fair to have a supplementary Register for children's trained nurses unless there can be some protection for the general trained nurse?" Now the whole of our object—what we are working for—is to obtain that protection for the general trained nurse. (Applause.) Once we have got the College of Nursing, with its Register, recognised by Parliament and given State protection—in other words, once we have got State Registration and the protection of the properly trained nurse—then it will be quite easy to deal with other matters such as supplementary Registers. The first matter of importance is to safeguard and to secure the position of the trained nurse. (Hear, hear.) Once that is done we can deal with the others later on. (Applause.)

The next is not so much in the nature of a question as of a statement: "There is a strong feeling among Poor-Law nurses that they should have more professional representation on the Council. It is constantly pressed on them by the Poor-Law Officers' Association that there are only three matrons on the Council who have Poor-Law experience, and that they were all trained in general hospitals." Well, I have got nothing to do with that as a statement, but, as I mentioned in my speech to the annual meeting, there are three very eminent ladies representing the Poor-Law nurses on the Council, and the actual representation to which the Poor-Law nurses are entitled at this moment is only four. The fourth representative will be appointed (see Speech, p. 325).

The Membership Standard.

This is another statement: "Many nurses are anxious that the standard of training should not be lowered any further. Is this at all likely to happen? Some nurses think that admission to the Register of the College is already becoming too easy." Well, I do not think there is the slightest possibility or likelihood of the standard of nursing being lowered by the College. As Miss Musson has told you, one of our principal objects is to raise the standard in every possible way. As regards admission to the Register being too easy, I can only tell you my own experience. Being a mere man, and not accustomed to deal with these matters, I have thought that two or three nurses who were excluded from the Register had some fair claims to be on it. I put forward those claims, tentatively of course, and with proper modesty (laughter), and I can only tell any of those who think that admission to the College Register is being made too easy, I wish they had been there when I did it. (Renewed laughter.)

Agreement on State Registration.

Here is another question: "Is it not possible to arrive at a reasonable agreement with the State Registration Society in order that full co-operation may be taken advantage of to bring about the end that both parties desire—*i.e.*, the State Registration of Nurses?" I repeat what

### THE COLLEGE OF NURSING, Ltd.—(*continued*).

### Important Questions and Clear Answers.

I said before, that we did everything in our power to arrive at an agreement with the Committee for State Registration. As they chose to break off negotiations, it is for them to ask for their resumption. We have already told them that if they wish, or if any other Society which can claim to represent nurses wishes, to confer with us with the object of getting State Registration, we are only too happy to meet them. I believe very strongly myself that if we lose this opportunity for getting State Registration it may not occur again for many years to come. I have also got a very strong feeling that the nursing profession may find itself up against considerable difficulties and considerable dangers if it does not carry through State Registration before the end of the war. I believe myself that if we were to combine now, if we could get these people, some of whom, I am afraid, are working rather to serve their own selfish ends, to combine with us and come forward with an agreed Bill, we could get it within a very short space of time. (Applause.) But it is absolutely impossible to negotiate with people who, while agreeing to negotiate, are openly and secretly doing their absolute best to damage the people with whom they pretend to be working. (Applause.)

#### The Poor-Law Nurse.

The question which is next put is: "Why should the question of Poor-Law trained nurses arise at all? If trained in satisfactory training schools, these nurses seldom have any connection with the Poor-Law Officers' Association." Well, I am very sorry that the question of representation of any one section of nurses has arisen at all. We have never introduced that idea. We have never recognised the slightest difference between one nurse on our Register and any other nurse. The minute a nurse is on our Register it means that she has a proper certificate from a properly recognised school, and we ourselves have never asked whether she was trained in a Poor-Law or any other school. That is the spirit I should like to see prevail, and that is the spirit that I hope eventually will prevail. (Applause.)

